# Clinical features, management, and prognosis of *Bacillus cereus* sepsis in premature neonates

**DOI:** 10.1097/MD.0000000000034261

**Published:** 2023-07-14

**Authors:** Na Li, Yunlin Shen, Xiaohui Gong, Wenchao Hong, Juan Li, Hongzhuan Zhang

**Affiliations:** a Department of Neonatology, Shanghai Children’s Hospital, Shanghai Jiao Tong University, Shanghai, China.

**Keywords:** *Bacillus cereus*, neurodevelopment, premature, prognosis, sepsis

## Abstract

This study aimed to investigate the clinical characteristics, management and prognosis of *Bacillus cereus* sepsis in premature neonates. The clinical information of 8 premature neonates with *B cereus* sepsis who were treated in Shanghai Children Hospital from January 2015 to December 2019 was retrospectively collected from the medical records and analyzed. The neurodevelopment related conditions were collected at follow up visits at corrected age of 6 months and 12 months. Five patients developed meningitis, and cerebral magnetic resonance image showed abnormal in 5 patients. After treatment with meropenem and vancomycin, 1 patient died, and 7 patients survived and were smoothly discharged. At follow up visits, 1 patient was diagnosed with hydrocephalus and showed severely delayed neurodevelopment, 2 patients had mild delayed neurodevelopment, and the neurodevelopment was basically normal in remaining 4 patients. *B cereus* infection can cause severe complications of central nervous system, and affect neurodevelopmental outcome. Antibiotic treatment with meropenem and vancomycin is proven to be effective. Refreshing the central catheters is helpful for the prevention of *B cereus* sepsis and cerebral magnetic resonance image may be employed for the prognosis assessment.

## 1. Introduction

*Bacillus cereus* is a type of Gram positive, aerobic facultative bacillus which is widely found in the environment.^[1]^ *B cereus* is a spore forming and ubiquitous bacterium present in soil, food, insect larvae, almost all surfaces and human skin.^[2,3]^ The *B cereus* group is comprised of 8 closely related species: *B cereus, Bacillus cytotoxicus, Bacillus mycoides, Bacillus pseudomycoides, Bacillus thuringiensis, Bacillus weihenstephanensis, Bacillus toyonensis*, and *Bacillus anthracis*.^[4–6]^ In adults, *B cereus* mainly causes gastrointestinal infection and has been the third most common cause of food poisoning.^[7]^ Besides food poisoning,^[8]^ *B cereus* can also cause focal or systemic infection, including sepsis, endophthalmitis, pneumonia, meningitis and encephalitis, especially in immunosuppressed patients, and delayed treatment often compromises the clinical outcome. Contaminated ventilator equipment, intravenous catheters and ventriculoperitoneal shunts have also been identified as routes of transmission in neonates with *B cereus* meningitis.^[9,10]^

Neonatal sepsis is a serious disease which threatens the health and life of neonates. The incidence of neonatal sepsis is 4.5‰ to 9.7‰ among survived neonates^[11]^ and increases as gestational age and body weight decrease.^[12]^ The term and/or premature neonates are susceptible to late-onset sepsis. Common pathogens for neonatal late-onset sepsis in China are *Staphylococcus aureus, Pseudomonas aeruginosa*, and *Klebsiella pneumoniae*. Though *B cereus* is a rare cause of neonatal sepsis, it can also cause hemorrhagic meningoencephalitis which may severely damage the developing brain of the neonates. Recently, some studies have reported *B cereus* infection in premature neonates, but the neurological outcome of these patients is poorly understood. This study was to analyze the clinical characteristics and management of *B cereus* infection in 8 premature neonates as well as the prognosis at 6 and 12 months, and the effects of empirical antibiotic therapy on the clinical outcomes of *B cereus* infection were also explored.

## 2. Methods

### 2.1. Ethical statement

This study was approved by the Ethics Committee of Shanghai Children Hospital (2021R036-E01).

### 2.2. Participants

We screened preterm neonates with sepsis who were hospitalized in the Department of Neonatology, Shanghai Children Hospital from January 2015 to December 2019. Eight premature neonates were diagnosed with *B cereus* sepsis based on the clinical manifestations and results from blood culture (After disinfection, 1 mL of blood was collected for blood culture).

### 2.3. Data collection

The information about clinical features and treatments was retrospectively collected from the medical records, and the prognosis was evaluated at follow up visits.

### 2.4. Outcome

After discharge, we followed up the patients, the patients received Gesell Developmental Schedules scoring at 6 months of corrected age and 7 patients completed Ages & Stages Questionnaires (3rd Edition) (ASQ-3) at 12 months of corrected age.

## 3. Results

### 3.1. General information

There were 6 males and 2 females. Cesarean section was noted in 4 patients and vaginal delivery in remaining 4 patients. The gestational age at birth was 29 to 35^+4^ weeks; the birth weight ranged from 1060 g to 2330 g; the Apgar score at 5 minutes was 8’-10’; the age at the diagnosis of sepsis ranged from 4 days to 31 days; 5 patients (No 1, 2, 4, 6, and 7) had a history of premature prolonged rupture of membrane; 1 patient (No 3) had a record of maternal cervical incompetence; 2 patients (No 5 and 7) were smaller than gestational age, 1 patient (No 2) received anti-infective treatment before delivery because her mother had positive blood culture. The main clinical features are summarized in Table 1.

**Table 1:** Characteristics of neonates with Bacillus cereus infection.

Case	Gestational age (wk)	Birth weight (g)	Gender	Delivery pattern	Age of sepsis (d of life)	Clinical features	Treatment	Anti-infection time (d)	Outcome
1	29	1060	Male	SVD	30	Fever, apnea, abdominal distension	Intubation, meropenem, vancomycin	30	Survived
2	30 + 4	1590	Female	SVD	19	Apnea	CPAP, meropenem, vancomycin	14	Survived
3	34 + 1	2330	Male	SVD	7	Fever, convulsion, hypotension, metabolic acidosis	Intubation, meropenem, IVIG, fluid resuscitation, dopamine, dobutamine	3	Deceased
4	30 + 5	1660	Male	Cesarean section	10	Apnea, abdominal distension	CPAP, meropenem, IVIG	49	Survived
5	35 + 3	1470	Male	Cesarean section	31	Fever	Meropenem, vancomycin, IVIG	25	Survived
6	32 + 4	1630	Male	SVD	22	Fever, apnea	Meropenem, vancomycin, IVIG, dopamine, CPAP	26	Survived
7	34	1500	Male	Cesarean section	4	Fever	Meropenem, vancomycin, IVIG	39	Survived
8	35 + 4	2030	Female	Cesarean section	21	Fever	Meropenem, vancomycin, IVIG	30	Survived

CPAP = continuous positive airway pressure, IVIG = intravenous immunoglobulin, SVD = spontaneous vaginal delivery.

### 3.2. Clinical manifestations

Six patients (No 1, 3, 5, 6, 7, and 8) had fever with the highest body temperature at 38.2°C to 38.8°C; none developed hypothermia; 1 patient (No 3) presented with convulsions; 4 patients (No 1, 2, 4, and 6) developed apnea; 2 patients (No 2 and 7) had abdominal distension and feeding intolerance; 1 patient (No 3) refused feeding; 1 patient (No 2) was on non-peros due to suspicion of necrotizing enterocolitis. As shown in Table 2, after birth, 1 patient (No 1) received mechanical ventilation with continuous positive airway pressure for 3 days; 4 patients (No 5, 6, 7 and 8) had indwelling of peripherally inserted central catheter (PICC) for 7 to 27 days, and the PICC was removed after the sepsis resolved; 3 patients (No 1, 2, and 4) had peripheral venous catheter; 1 patient (No 3) had no peripheral venous catheter. All patients had positive blood culture for *B cereus*.

**Table 2:** Time of mechanical ventilation and catheter indwelling.

Case	Mechanical ventilation (d)	CPAP (d)	Peripheral venous catheter (d)	PICC (d)
1	3	27	30	0
2	0	2	19	0
3	0	0	4	0
4	0	8	10	0
5	0	2	5	27
6	0	7	15	7
7	0	0	1	7
8	0	0	13	9

CPAP = continuous positive airway pressure, PICC = peripherally inserted central catheter.

### 3.3. Treatments

Among these patients, 5 (No 1, 2, 3, 4, and 6) received respiratory support; 2 (No 1 and 3) were intubated and received mechanical ventilation; 3 (No 5, 7, and 8) received noninvasive ventilation; 2 (No 3 and 6) developed septic shock, and received fluid resuscitation and inotrope treatment; 5 (No 4,5,6,7,8) received intravenous infusion of immune globulin for immune support therapy.

Based on blood culture results, vancomycin was administered at meningitis dosing. In 6 patients, the disease condition was improved after vancomycin treatment; 1 patient (No 2) was treated with meropenem and ampicillin; 1 patient (No 3) was initially treated with benzylpenicilin and ceftazidime and then with meropenem, but the neonate was non-responsive to treatment and died. The patients with clinical improvement received antibiotic therapy for 14 to 49 days (Table 1).

### 3.4. Laboratory examinations

In 2 patients (No 2 and 8), the White blood cell count and the neutrophil ratio increased; leukocytopenia was noted in 5 patients (No 1, 3, 4, 5, and 6); 2 patients (No 3 and 7) developed thrombocytopenia; all the patients had increases in C reactive protein and procalcitonin to different extents. According to CLSI standard, all *B cereus* strains were resistant to penicillin and compound trimethoprim, but sensitive to erythromycin, clindamycin, gentamicin, vancomycin and levofloxacin. All the patients received lumbar puncture; 5 patients had concomitant meningitis according to the cerebrospinal fluid (CSF) assay (Table 3). The culture of all catheter tips failed to yield any bacterial growth.

**Table 3:** Results of laboratory examinations.

Case	Blood			CSF				MRI
	WBC (×109/L)	PLT (×109/L)	CRP (mg/dL)	WBC	Glucose (mmol/L)	Protein (mg/dL)	Time to normal CSF results (d)	
1	5.42	180	139	2700	0.6	5510	45	Hydrocephalus
2	34.76	209	26	2	2.9	1490	-	-
3	2.69	39	18	48000	12.1	62500	-	-
4	4.11	450	9	147	2.8	1760	40	Abnormal white matter signal
5	4.62	111	29	19	2	1610	10	Normal
6	4.08	443	8	153	1.9	2600	31	Abnormal signals in the lateral ventricles
7	8.1	65	40	98	1.8	2480	15	Cerebromalacia
8	18.11	304	28	18	2.3	1480	3	Meningeal enhancement

CRP = C reactive protein, CSF = cerebrospinal fluid, MRI = magnetic resonance image, WBC = White blood cell count.

### 3.5. Cerebral magnetic resonance image (MRI)

**Figure 1.**
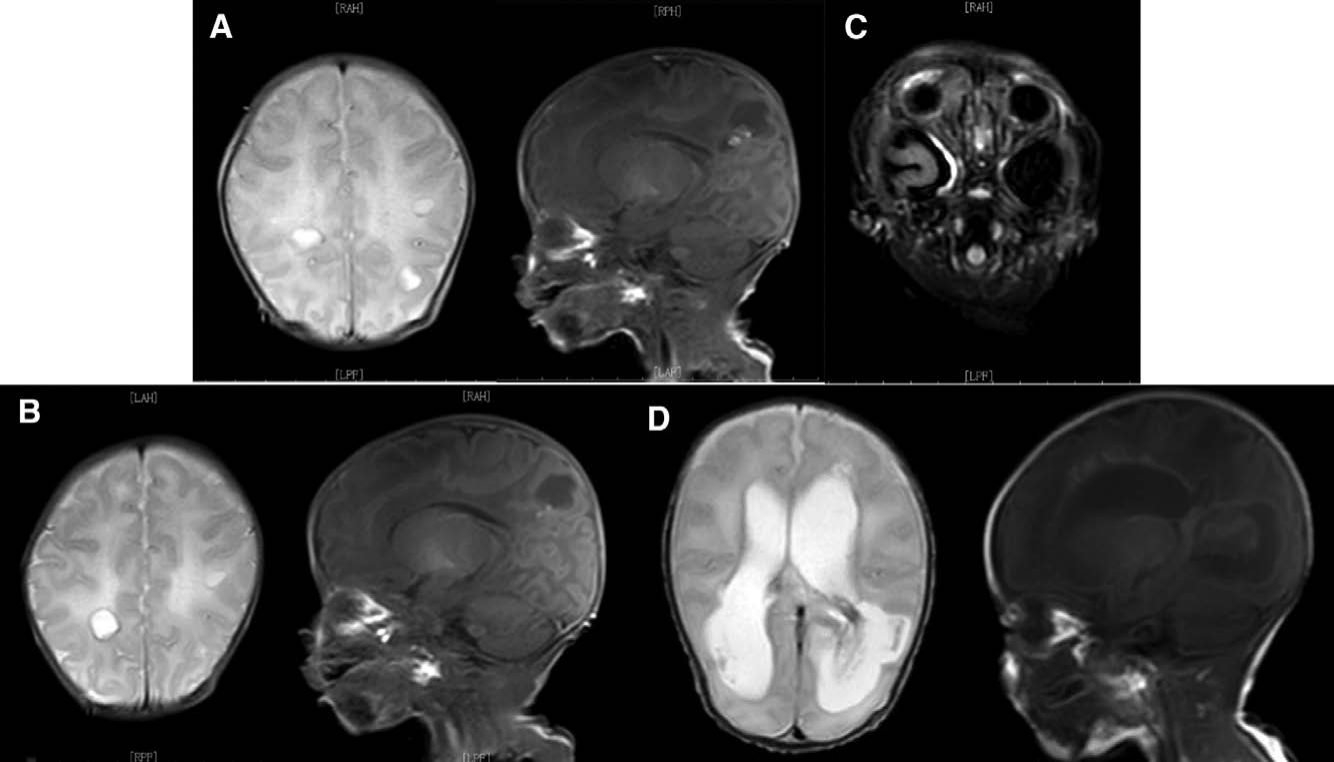
Cerebral MRI. (A and B) Hemorrhagic liquefactive necrosis of left hemisphere. (A) Abnormal hyperintensity at frontoparietal temporal lobes on both sides; T1WI sagittal view mixed low point hyperintensity, and hyperintensity on T2WI. (B) Reexamination after 2-wk treatment. Multiple patchy abnormal hyperintense foci at frontoparietal temporal lobes on both sides, with similar range to that in A; spotted hyperintensity on both T1WI and hypointensity on T2WI; while there was no significant change where T2WI had hyperintensity and T1WI had hypointensity (softening lesions). (C) T2-flair enhancement. Right temporal meningeal enhancement. Obvious enhancement (about 2.8 cm) was observed at the right temporal meninx. (D) Encephalocele and parietal lobe on both sides showed platy low T1 signal and high T2 signal, with ill-defined borders, partially connected to the rear corners of encephaloceles on both sides; encephaloceles on both sides were enlarged, while diocoel and epicotyl had no obvious enlargement. MRI = magnetic resonance image.

Six patients received cerebral MRI. As shown in Figure 1, MRI showed normal in 1 patient (No 5); 4 patients (No 1, 4, 6 and 7) had presentations of intracranial hemorrhage or leukomalacia; 1 patient (No 8) had meningeal enhancement on MRI; 1 patient (No 1) developed hydrocephalus on follow-up examination, and ventriculoperitoneal shunt operation was performed.

### 3.6. Outcomes

One patient died, and 7 patients survived and were discharged smoothly. All the survived patients received follow up through regular outpatient visit, the development of central nervous system was assessed and early intervention was administered if necessary. Four patients received Gesell Developmental Schedules scoring at 6 months of corrected age and all the patients completed Ages & Stages Questionnaires (3rd Edition) (ASQ-3) at 12 months of corrected age. Results showed 1 patient (No 1) had severely delayed neurodevelopment and underwent rehabilitation treatment, 2 patients (No 5 and 6) had mildly delayed neurodevelopment, and the neurodevelopment was basically normal in remaining 4 patients (Table 4).

**Table 4:** Prognosis of central nervous system development.

Case	Gesell developmental schedules (6 mo)					ASQ3 (12 mo)				
	Social skill	Adaptability	Language	Gross motor	Fine motor	Communication	Gross motor	Fine motor	Problem solving	Personal-social
1	77	78	85	76	82	5	10	15	10	10
2	101	93	96	97	94	25	30	50	45	35
3	/	/	/	/	/	/	/	/	/	/
4	/	/	/	/	/	25	45	60	50	35
5	96	97	103	100	115	30	35	60	50	50
6	90	82	96	106	90	15	30	55	25	20
7	96	116	107	88	107	25	35	30	50	30
8	/	/	/	/	/	35	45	35	40	30

Gesell Developmental Schedules: Developmental quotient (DQ) ≤ 85 means abnormal.

ASQ3 Critical value: Communication 15.64, Gross motor 21.49, fine motor 34.50, Problem solving 27.32, Personal-social 21.73.

## 4. Discussion

*B cereus* has been found in a variety of environmental reservoirs such as ventilator equipment, intravascular catheters and linen. In addition to food poisoning, *B cereus* may also cause systemic or focal infections in immunologically compromised or immunocompetent individuals. The spectrum of infections includes fulminant bacteremia, central nervous system infection (meningitis and brain abscess) and other complications.^[1]^ The pathogenicity of *B cereus* is closely related to the production of tissue damaging/reactive exoenzymes. The secretin includes 4 kinds of hemolysins, 3 distinct phospholipases, an emesis-inducing toxin, and 3 pore-forming enterotoxin: hemolysin BL, nonhemolytic enterotoxins and cytotoxin K.

### 4.1. Clinical manifestations

The immune function of premature infants is not mature, and the incidence of sepsis is high. The clinical manifestations were atypical. *B cereus* is not a common bacterium associated with neonatal sepsis. Studies have reported that the majority of strains (41%) are isolated from neonates, 3/4 of whom were premature infants with low birth weight.^[13]^ The virulence test of bacterial strains has indicated that the average production of bacterial toxin from neonates is low, suggesting that neonates are particularly sensitive to *B cereus* infection, even the strains with low toxin production, or some other unknown factors may be responsible for the infection in neonates. Neonates are more susceptible to *B cereus* infection than adult, especially in those with catheter indwelling. In our study, all the neonates were preterm and had venous catheter indwelling after birth, and half of them had PICC. In our cases, the culture of all catheter tips failed to identify any bacteria. However, catheter indwelling is a risk factor of *B cereus* infection. Thus, we recommend that once *B cereus* infection is confirmed in children, the catheters should be removed as soon as possible, and the catheters may be indwelt again once the infection is controlled after effective antibiotic therapy. In addition, *B cereus* can cause severe late-onset hemorrhagic meningoencephalitis in preterm infants. In our study, we found that the clinical manifestations of these 8 patients were more like those of gram-negative sepsis. 6 patients had fever, 4 patients developed apnea, 1 patient presented with convulsions. These signs can also be initial signs of neonatal meningitis, especially convulsion.

### 4.2. Clinical examinations

According to the evaluation for neonatal sepsis,^[14]^ for newborns with sepsis, especially those with central nervous system symptoms, the lumbar puncture should be further improved. In our study, all patients received lumbar puncture; 5/8 neonates had signs and symptoms of meningitis (CSF assay showed leukocytosis, low glucose, and increased protein). Lequin et al found *B cereus* could cause a severe late-onset hemorrhagic meningoencephalitis in preterm infants, the ultrasound and MR images showed a typical pattern of mainly hemorrhagic and early cavitating, selective white matter destruction.^[15]^ According to the patient symptoms and the results of lumbar puncture, 6/8 patients received cerebral MRI examination, among whom 5 had abnormal findings on cerebral MRI. 1 patient had normal CSF assay but abnormal findings on MRI. We, therefore, believe that lumbar puncture and cerebral MRI are necessary for preterm infants with *B cereus* septicemia.

### 4.3. Clinical treatments

Some studies have reported that most *B cereus* strains are resistant to penicillin and cephalosporin, but sensitive to aminoglycoside, carbapenem, vancomycin and chloramphenicol.^[16]^ In our study, the patient was initially treated with benzylpenicilin and ceftazidime and then with meropenem, but the neonate was non-responsive to treatment and died. The other 7 patients were first treated with meropenem for empirical anti-infective therapy, which was effective, and vancomycin or ampicillin sulbactam were added according to the results of drug susceptibility tests. Our results showed *B cereus* strains were responsive to meropenem or vancomycin.

### 4.4. Outcomes

Preterm infants themselves are at risk for poor nervous system development, and sepsis and encephalopathy can increase the risk of stunting.^[17]^ The *B cereus* septicemia in preterm infants is often serious and high mortality, and these patients are more likely to have neurological sequelae. Our study showed the prognosis was acceptable in 4 out of 7 surviving preterm infants (4/7) after active intervention, which was largely related to the close follow-up and good compliance to treatments. In the follow-up period, clinicians should also pay more attention to the development of the nervous system in the later stage, and timely intervention should be carried out for premature infants with *B cereus* septicemia if necessary.

### 4.5. Limitations and experience

There were still limitations in this study. In our study, only PICC tips were sampled, followed by bacterial culture, but breast milk, formula milk, sheets and other substances were not sampled for bacterial culture. Thus, the source of infection is unknown. The concentrations of vancomycin and meropenem were not detected in the CSF. This was a retrospective study, and the sample size was small. In short, the clinical manifestations of *B cereus* sepsis in premature neonates are nonspecific, serious central nervous system injury may be present, and the developmental outcome is usually poor. On laboratory examinations, C reactive protein increased, CSF examination showed features of meningitis, and hemorrhagic meningoencephalitis was observed on cerebral MRI. Preterm labor, mechanical ventilation, and intravenous catheter indewelling may be high risk factors for *B cereus* infection. Meningitis dosing meropenem and vancomycin are proven effective in the treatment of *B cereus* infection. According to our experience, the central catheters should be removed, if necessary. For the preterm infants with *B cereus* septicemia, clinicians should pay attention to the development of the nervous system at late stage, and timely intervention should administered if necessary. Of note, more studies with large sample size are warranted to confirm our findings.

## 5. Conclusions

Positive blood culture of *B cereus* in premature neonates should not be deemed as contamination. *B cereus* infection can cause severe central nervous system complications. Lumbar puncture should be performed and timely anti-infective treatment is usually indispensible. Meningitis dosing meropenem and vancomycin are often effective in the treatment of *B cereus* infection. Cerebral MRI is needed before the antibiotic treatment is discontinued. Clinicians should pay attention to the neurological development at late stage for preterm infants with *B cereus* septicemia.

## Author contributions

**Conceptualization:** Na Li.

**Data curation:** Na Li.

**Formal analysis:** Na Li.

**Investigation:** Na Li, Yunlin Shen, Xiaohui Gong, Wenchao Hong, Juan Li, Hongzhuan Zhang.

**Methodology:** Na Li, Yunlin Shen, Xiaohui Gong, Wenchao Hong, Juan Li, Hongzhuan Zhang.

**Writing – original draft:** Na Li.

**Writing – review & editing:** Yunlin Shen.
